# Development of a toolkit to help parents/caregivers manage feeding problems in autistic children: A protocol for a realist synthesis and toolkit co-design

**DOI:** 10.1371/journal.pone.0309410

**Published:** 2024-10-16

**Authors:** Zoe L. Connor, Lou Atkinson, Rachel Bryant-Waugh, Ian Maidment, Jacqueline Blissett

**Affiliations:** 1 Institute of Health and Neurodevelopment & School of Psychology, Aston University, Birmingham, United Kingdom; 2 Department of Nutrition and Dietetics, University Hospitals Coventry and Warwickshire NHS Trust, Coventry, United Kingdom; 3 School of Psychology, Aston University, Birmingham, United Kingdom; 4 Warwick Medical School, University of Warwick, Warwick, United Kingdom; 5 Maudsley Centre for Child and Adolescent Eating Disorders, South London and Maudsley NHS Foundation Trust, London, United Kingdom; 6 Department of Child and Adolescent Psychiatry, Kings College London, Institute of Psychiatry, Psychology and Neuroscience, London, United Kingdom; 7 Aston Pharmacy School, Aston University, Birmingham, United Kingdom; Public Library of Science, UNITED STATES OF AMERICA

## Abstract

Many autistic children have feeding problems, typically eating a limited range of foods. Feeding problems affect quality of life, health, and development. Research suggests that parents are often unsure when to or whether to seek help. When they do, local provision of help across the UK is often lacking. A toolkit could offer a tailored, accessible, and scalable early intervention to support parents. We aim to develop the blueprint of a toolkit to help parents/caregivers manage feeding problems in their autistic children. Medical Research Council guidance on developing complex interventions informs three successive work packages:

1. Realist review: a literature search and analysis using realist theory of logic to construct programme theory(s) in line with RAMESES (Realist And Meta-narrative Evidence Syntheses: Evolving Standards) guidance.
2. Realist evaluation: interviews of three groups: autistic children, young people and adults (*experts*), parents/caregivers of autistic children (*parents*), and professionals who help parents manage feeding problems (*professionals*) across the UK. Analysis of verbatim interview transcripts using realist theory of logic to refine programme theory(s).
3. Co-design of the toolkit blueprint: behaviour change theory applied to the programme theory(s) will generate candidate components for the online tool. A blueprint (a detailed textual outline) will be co-designed.

A participatory research team of *experts*, *parents*, and *professionals* will be involved in each work package. Where consensus is needed it will be reached by asynchronous nominal group technique. A PPI (public and patient involvement) advisory group of *experts* and *parents* will ensure the project is relevant, respectful, and accessible. Findings of each step will be disseminated via journal publications, conferences, social media, as well as PPI-co-produced webinars and a dissemination event. On completion, this project will provide the foundation for the subsequent development and refinement of the prototype toolkit.

## Introduction

Feeding problems in autistic children are common, stressful, and damaging to children’s health and development. Autism is a condition characterised by differences in social interaction and behaviour [1]. This proposal adopts the autistic community’s preferred terminology: autism (not autistic spectrum disorder) and autistic people (not people with autism) [2, 3].

Feeding problems are “eating-related behaviours that are unrelated to weight, shape, and/or body image concerns, yet impair functioning” [4, 5]. Feeding problems are five times more common in autistic children compared to non-autistic children and often lead to narrow food repertoires [6].

Reasons for high rates of feeding problems in autistic people are poorly understood [5]. Eating is an unpredictable sensory-rich experience intertwined with complex social rituals; however, combinations of autistic preferences for sameness, social difficulties, and common sensory processing differences likely make the eating experience stressful rather than pleasurable [7].

Feeding problems impact a child’s and their family’s physical and mental health and lead to a poor quality diet [8]. Autistic children are more likely to have micronutrient deficiencies; sometimes with life-threatening or life-changing consequences e.g. vision loss [9–11]. In interviews, parents of autistic children described mealtimes as “one of the most stressful times of the day” [12]. Dietary patterns and preferences laid down in early life often persist to adulthood [13] and therefore targeting feeding problems in childhood promotes lifelong healthier diets. However, many parents are unsuccessful in getting NHS support for diet and nutrition issues, and when they do, they do not find the support offered helpful [14, 15].

Addressing feeding problems in autistic children could reap many rewards; an improvement in nutritional status could improve learning, behaviour, mental and physical health, improve life expectancy, and reduce reliance on social care. It could reduce parental stress, promoting their physical and mental health too. Numerous distressing and costly impacts of feeding problems may be prevented by an early, accessible intervention: hospital admissions due to malnutrition, tube-feeding, intensive feeding therapy, and supports needed after the irreversible damage of micronutrient deficiencies (e.g., vision-loss) [16–18]. This research aims to develop a toolkit to help parents manage feeding problems in autistic children—which will promote lifelong mental and physical health through healthier diets.

## Methods

### Aim

To develop a toolkit that helps parents/caregivers (hereafter parents) manage feeding problems in autistic children and young people (hereafter children); to improve their short-term and long-term health, development, and quality of life.

### Objectives

1. To conduct a realist review to identify important components for the toolkit and to understand the mechanism, context, and outcome of successful interventions to result in the co-creation of programme theory(s)
2. To conduct a realist evaluation via interviews of *experts*, *parents* and *professionals* to test, refine and finalise the programme theory(s)
3. To co-design the blueprint of a toolkit, based on the programme theories emerging from the realist review and evaluation.

## Project plan

The toolkit development is informed by the Medical Research Council (MRC) guidance for complex intervention development [19–21] and is rich in patient and public involvement.

### Patient and public involvement

Patient and public involvement in intervention development ensures that it truly benefits those it is being designed for. This project idea came from discussions with parents and professionals over many years of the first author’s clinical practice highlighting the significant gaps in provision by the NHS across the UK and bolstered by the findings from their qualitative study of parents of autistic children with feeding problems (20). Three members of the public were further consulted in the finalisation of this protocol: one non-autistic parent of an autistic child, one autistic parent of an autistic child and one autistic adult. Two groups including autistic children and adults have been convened to assist in this research: a PPI advisory group and a participatory research team. All members of these teams reside in the UK.

The PPI advisory group comprises three parents of autistic children and three autistic people has been convened. The PPI group advises on the relevance, respectfulness, and inclusivity of the project plan, participant information, and interview schedules and will co-produce dissemination materials and co-host dissemination events.

The participatory research team comprises three autistic young people and adults who have had feeding problems (*experts*), three parents of autistic children who have or have had feeding problems (*parents*), and three professionals who help autistic children and their families with feeding problems (*professionals*). At least half the group are autistic. The team aids the primary researcher throughout the project with making sense of findings and co-designing the toolkit in the final stages.

Contributors for each group were recruited purposively via social media, the National Autistic Society, support groups and/or personal contacts, with specific calls for autistic people using different communication modes, autistic people with intellectual disability, and autistic people and/or parents from Black and minority ethnic communities. Meetings are carried out online at least three times a year via Microsoft Teams video call with the option for asynchronous participation. Guidelines that were coproduced by AASPIRE (Academic-Autistic Spectrum Partnership in Research and Education) have been adapted to ensure that meetings are accessible and to address power differences [22]. All contributors are reimbursed for their involvement in line with National Institute of Health and Care Research (NIHR) guidance [23]. Training in research methods is carried out when necessary.

Nominal group technique (NGT) [24] will be used to obtain consensus from the participatory research team when needed. Asynchronous NGT ensures accessibility for those who have slow processing speeds, need to use assisted communication, or who may find face-to-face/online synchronous group discussions and more complex consensus techniques challenging. It also reduces power differentials that can skew a consensus process when being carried out face-to-face where for example professional voices may dominate [22, 24].

NGT will be carried out in the following steps:

1. Options that need to be chosen are presented to the group via video and/or text/email.
2. Group members individually comment on the content using an online collaborative tool (e.g. Padlet).
3. Group members group content on the importance of inclusion.
4. Primary researcher re-presents findings.
5. Group members rate each piece of previously selected content.
6. The process continues until a final agreement has been reached [24]

### Work packages

The toolkit will be a complex intervention [19]. Its development is informed by the Medical Research Council (MRC) guidance for complex intervention development [19, 20, 25]. There are three successive work packages. Work package (WP) 1 involves a literature review using a realist approach—a realist review, WP2 involves qualitative interviews using a realist approach—a realist evaluation. WP3 is the codesign of the toolkit blueprint.

**WP1: A realist review.** *Aim*. To identify the evidence base and develop programme theory(s)

*Objectives*.

1. To conduct a realist review to identify the mechanism, context, and outcome of successful interventions.
2. To develop programme theory(s)

Research question: Interventions for feeding problems in autistic children. What works, for whom, under what circumstances, and why?

*Methods*. Complex interventions must have a coherent theoretical basis to be an intervention worth implementing and to ensure the best return on investment [19, 21, 25]. Realist approaches aim to examine what works, for whom and in what circumstances and why. Realist approaches are increasingly popular in the development and evaluation of healthcare due to their pragmatism, flexibility and likelihood of generating findings that can be applied in real-life settings than more traditional positivist or constructivist approaches [26–30].

The steps below are incremental and iterative. They are in line with RAMESES (Realist and Meta-narrative Evidence Syntheses: Evolving Standards) [31], and the successful realist synthesis related to medicine management in older people (MEMORABLE) [29, 32].

Steps.

1. Review registration: with PROSPERO (the NIHR International prospective register of systematic reviews) [33].
2. Background search: A background search used key words (autis* and feeding problems) in the internet search engine Google. Results were sampled to find a few each of first-hand accounts, guidance from health bodies and charities, and academic papers. The aim of this search was to take a small broad sample of available records to begin theory generation.
3. Data extraction:
  - Record information: Descriptive characteristics of the records yielded in the background search were entered into an Excel spreadsheet: i. Publication details (author/title/date) ii. Record type (study/review etc) iii. Study and sample details where relevant (sample size, ages, genders, ethnicity, location) iv. Outcomes where relevant.
  - Rating of records: Each record was rated in the Excel spreadsheet according to their relevance, richness and rigour in line with realist synthesis convention [34].
  - Coding: Full text of records deemed to be relevant and rich enough for use in the review were be imported into the qualitative data analysis software NVivo14 (QSR International). The full text records were coded into nodes that identified interventions, contexts, mechanisms, and outcomes.
4. Theory generation: Examination of the different buckets of coded data generated ‘hunches’ of initial programme theory(s). These briefly worded hunches were then created as nodes on NVivo, and the records recoded into these. A memo was created for each ‘hunch’ node that will be used to document the evolution of each emerging initial programme theory as per the suggested use of NVivo in [35]. The initial theories were fleshed out within these memos to be in the form of very rough context, mechanism, outcome configurations.
5. Systematic search: An information specialist trained in realist searching has been recruited onto the research team and involved in team meetings to ensure she has a good understanding of the project. The information specialist designed an initial search strategy which was then piloted and adjusted with the aim for the search to yield representative.
6. Screening and sifting: Records from this initial systematic search are being imported into Rayyan [36] for screening for inclusion based on title and abstract indicating relevance against inclusion criterion. Excluded studies, and the rationale for exclusion, are being recorded.
7. Data extraction: The steps in 4a) record information and 4b) rating of records will be carried out for included records. Full text of records deemed to be relevant rich and rigorous enough for use in the review will be imported into NVivo14 (QSR International), and coding carried out using realist theory of logic to enable programme theory to emerge. Coding is inductive (from the data), deductive (from interpretations of the emerging theory), and retroductive (interpretation of data for deeper hidden causal forces). Codes cover concepts that are important and potentially relevant to the programme theory(s).
8. Programme theory(s) consultations: emerging theories will be developed and refined in meetings with the research team and participatory research team. They will be in the form of Context-Mechanism-Outcome configurations (CMOCs) i.e., in these circumstances, for these people, this works, because of this [37] (Data analysis and synthesis: Programme theory will be generated and refined using a realist logic of analysis, the building of context-mechanism-outcome configurations (CMOC) that describe how contextual factors (C) trigger particular mechanisms (M) to generate various outcomes (O) and therefore to understand how an aspect of an intervention works or might be expected to work in what conditions [37].
9. Iterative searching: Phased and differently focused searches will be carried out:
  - In line with realist review guidance, searches will be purposive, iterative, and theory driven.
  - Search strategies will be developed using the CIMO (Context, Intervention, Mechanisms, Outcome) question framework, to continue to construct a plausible, coherent programme theory.
  - Searches may include academic databases (MEDLINE, SCOPUS and CINAHL), CLUSTER searching (i.e., iterative searching using Citations, Lead authors, Unpublished materials, Scholar searches, Theories, Early examples, and Related projects), grey literature (theses, guidelines, reports, toolkits, policy documents, websites of relevant organisations and charities) via targeted google searches, theses repositories (https://ethos.bl.uk/ and https://oatd.org/) and grey data (social media posts, blog posts, podcasts) via targeted google searches, site-specific searches on X and Reddit.
  - There will be no limitations on the record types (including study designs in academic papers) to be included, nor language nor date restrictions.
  - Each round of searching will cycle through steps 7 to 9: screening and sifting; data extraction; programme theory consultations.
  - At each stage of data synthesis juxtaposition, reconciling, adjudication and consolidation of data will be applied as needed.
  - This cycle will continue until adequate theoretical saturation is reached i.e., when new searches are no longer adding significantly to the development of programme theory(s) that can be tested and bolstered in work package 2.

## WP2: A realist evaluation

*Aim*. To test and refine the programme theory(s)

*Objectives*. To conduct interviews with *experts*, *parents*, and *professionals* to test and refine the programme theory(s)

Research question: Interventions for feeding problems in autistic children. What works, for whom, under what circumstances, and why?

*Methods*. Based on the methodology used in the MEMORABLE study [29], realist-informed interviews will be carried out following these steps:

1. Interview schedules: Informed by WP1 findings, realist-informed interview schedules will be created to explore the research question. Interviews will be different for each group: A) *experts*: autistic children and young people (age 8–25), B) *parents*: parents of autistic children and young people (age 3–25), and C) *professionals*: professionals who help autistic children and young people with feeding problems. All participants will reside in the UK. Piloting of the interview schedules and accessibility checks will be carried out with the PPI advisory group.
2. Recruitment: purposive: via adverts through charities and social media; flyers handed out in clinics in NHS sites and schools that have been approved to be participant identifying centres (PIC); and snowball sampling. Care will be taken to involve participants with intersectionality of characteristics proffering higher risk of health inequalities e.g., those with intellectual disabilities and from Black, Asian, and Minority Ethnic (BAME) communities. Targeted inclusion of autistic people and their families from specific BAME groups is key to ensuring the toolkit captures factors useful for these groups. Differing feeding practices between ethnic groups from infancy result in different growth patterns predictive of different health outcomes [38–40]. Black, Pakistani and Bangladeshi adults experience higher rates of diet-related health inequalities that are not explained by other factors such as deprivation [41]. BAME autistic people and their families are underrepresented in research and often find services less accessible to them due to a lack of cultural competence [42, 43].
3. Interviews: will be held online via a mode and time chosen by participants as best for them, e.g., Microsoft (MS) Teams video, phone, text chat, face-to-face with assisted communication (if local). Participant information and consent forms will be provided to participants before the groups or interviews in accessible formats. Demographic information will be collected to evaluate the representativeness of the sample related to the identified equality, diversity, and inclusivity aspects. Interpreters and expert consultants will advise and help to enable full participation for those using assisted technology (e.g., speech and language therapists or autism education specialists). Questions will be given in advance of the interviews to enable those with processing differences or using assisted communication to formulate responses. Interviews will be recorded (by MS Teams/digital recorder as appropriate) and transcribed verbatim. Interviews will follow the schedule but be flexible to allow exploration of causal accounts as they emerge. They will take approximately an hour. The evidence-based visual communication aid Talking Mats^TM^ will be offered in each interview [44]. This involves the sorting of different picture cards under columns to aid in communicating views. Participants will be offered shopping vouchers to thank them for their involvement [23].
4. Sampling: Data will be analysed in batches after five interviews of each group are carried out and then continued until adequate depth and breadth of data has been collected (saturation achieved). It is estimated this will be around fifteen interviews in each group.
5. Analysis: Transcriptions will be coded in NVivo 14 using the same realist logic of analysis as in WP1. The interviewee data will be analysed and coded for the underpinning CMOCs.
6. Synthesis: The CMOCs from WP1 and WP2 will be synthesised, looking for consistent patterns and emerging programme theory(s). Findings will be discussed, and further iterations of analysis and literature searches undertaken, as necessary. Final programme theory(s) will be co-produced, with consensus reached via NGT.

## WP3: Toolkit co-design

*Aim*. To co-design a blueprint of the toolkit from the programme theory(s) generated in WP1 and WP2

*Objectives*.

1. To co-create a process model and outcomes of a toolkit by applying behaviour change theory to the programme theory
2. To co-design a blueprint of the toolkit from the emergent behaviour change candidates

*Methods*. The programme theory(s) developed in WP1 and WP2 will be used in this final stage of development to model the process and outcomes of the toolkit and a subsequent blueprint (outline). Secondary outputs will be the research publications as detailed in the dissemination section.

*Steps*.

1. Process and outcome modelling: Middle-range theory (e.g., behaviour change theory) will be applied to the emergent programme theory(s). The middle-range theory(s) chosen will depend on the findings: as the key principle of complex intervention design is flexibility and iteration [19]. A strong case has been made for the inclusion of behaviour change theory into complex intervention development [20, 45]. Decades of cross-discipline applied research has enabled the rigorous characterisation of the ‘active ingredients’ of interventions such as goal setting, graded tasks, instruction, and social supports into the Behaviour Change Techniques Taxonomy (v1) (BCTTv1) [46]. Ninety-three distinct Behaviour Change Techniques (BCTs) have been identified and mapped via expert consensus to theoretical constructs underlying behaviour change e.g., knowledge, goals, optimism (the Theoretical Domains Framework (TDF)) [47]. Having a common international language in which to describe behaviour change components of interventions is key for comparison, replication, and trials of successful components and combinations of components. By mapping the programme theory(s) to the TDF, candidate BCTs can be identified from the BCTTv1 which would be likely to facilitate change in the behaviours that will be needed to overcome feeding problems in autistic children. From these candidate BCTs, we will select the BCTs to be included and the mode of delivery for each BCT.
2. Blueprint co-design: Using the process and outcome modelling, a blueprint (a detailed outline e.g., textual descriptions and diagrams on a Word document) will be co-designed. This will involve the participatory research group and a web-developer or digital health intervention expert (as a digital element is likely). The NGT consensus process will be used, with iterative rounds until a finished product has been codesigned. The content and delivery of the toolkit will be decided following the stages in this project. It is envisaged that the toolkit will be best delivered either in its entirety or in conjunction with a website/app that is behind a log-in wall. The parent would complete questionnaires (e.g., validated nutrition and feeding screening tools) and then access content tailored to their answers. This content will be incorporated from existing sources or created for the tool, and take the form of videos, written content, and short training courses. Other components of the toolkit may be forums, ‘ask the expert’ sections, or gamification (badges, rewards). Tailored signposting to other services e.g., GP, will be given when considered necessary by scores on questionnaires.

## Ethical considerations

This project will be carried out in accordance with the Helsinki declaration, the Health and Care Professions Council Code of Conduct and NIHR and local ethical guidelines [48–51]. Production of participant information, collection of informed consent and appropriate debriefing will be carried out in line with NIHR and AASPIRE best practice guidance, and reviewed by the PPI advisory group [22, 50]. Verbal consent will be recorded if any participant is unable to provide written consent. Written parental consent will be obtained for all participants who are 16 and under, alongside assent from the child or young person. All sensitive data collected for this study will be treated confidentially and stored securely in accordance with the Data Protection Act 2018 [52].

Ethical approval from Aston University Research Ethics Committee (REC) (ref:HLS21134) has been granted to collect anonymised data via targeted searches of social media in work package 1, the realist review. Aston University REC approval (ref:HLS21009) was also granted to collect data from the PPI advisory group and participatory research team discussions prior to convening the groups. All contributors have been provided with participant information sheets and informed consent has been collected. For the realist interviews in WP2, NHS ethical approval is being sought via the integrated research application system (IRAS), due to the recruitment of participants involving NHS sites.

## Dissemination plan

The research findings will be disseminated via research publications, conference abstracts, social media and PPI-coproduced webinars and final dissemination event. We will seek further funding to finalise the toolkit as per the MRC framework: prototype production, feasibility testing, refining the toolkit; multiphase optimization strategy (MOST) testing of included elements to determine the most effective combination [53], trial and health economic analysis of the final toolkit.

## Supporting information

S1 File (PDF)
