# Supplementary material for: Development of a toolkit to help parents/caregivers manage feeding problems in autistic children: A protocol for a realist synthesis and toolkit co-design
Source: PLoS One. 2024 Oct 16;19(10):e0309410. doi: 10.1371/journal.pone.0309410 (PMC11482719; doi:10.1371/journal.pone.0309410)
Supplement: S1 File — (PDF) [file pone.0309410.s001.pdf]

NIHR | National Institute for Health Research

Summary

Reference Number	NIHR302307
Lead Applicant	Ms Zoe Connor
Research Title	Development of a toolkit to help parents/carers manage feeding problems in autistic children
Plain English Summary	**What is autism and what are feeding problems?**<br>Autism is a condition affecting how people see the world and how they behave. Feeding problems are problems with eating such as only eating a few kinds of foods.<br><br>**What are the aims of this project?**<br>To develop a toolkit. It will help parents/carers help their autistic children with their feeding problems.<br><br>**Why is the project important?**<br>Many autistic children have feeding problems. Feeding problems are stressful for them and their families. They can lead to a poor diet. Poor diets can affect a child's health and learning. They can lead to mental and physical health problems, or make existing problems worse. Poor diets are one reason autistic people often die earlier than non-autistic people. Research suggests that parents are often unsure when or if they should ask for help. When they do ask for help, they may find that there is no help for them from their local services. A toolkit could help. It could provide the right support for the right people anywhere and anytime.<br><br>**What are we going to do?**<br>We will develop a detailed written plan of what we want the toolkit to include. This toolkit will need to meet the needs of children and parents with many different issues. It is a 'complex intervention'.<br><br>A team will help with each step of the research, called the *Participatory Research (PaG) team*. It will consist of three each of:<br>• autistic young people and adults (*experts*)<br>• parents of autistic children (*parents*)<br>• professionals who help autistic children with feeding problems (*professionals*)<br><br>We will follow established steps to develop a complex intervention:<br>• **Step 1:** We will search journals and guidelines to see what others think helps with this issue, and why.<br><br>• **Step 2:** We will ask other people what they think helps with this issue. We will ask about 50 *experts*, *parents*, and *professionals*. We will draw diagrams to show what helps autistic children with feeding problems and why.<br><br>• **Step 3:** We will compare the diagrams from step 2 to *behaviour change theories*. Behaviour change theories explain what helps people change. From them, we will have ideas for what will work in the toolkit. We will write a detailed plan of the contents of the toolkit.<br><br>**What will the tookit be like?**<br>We imagine the toolkit will be available on a website or app. Parents will answer questions about common problems like sensory issues, or anxiety. They will watch videos and read about things that might help their child. What they see will depend on the answers they gave about their own child. Parents will write goals for themselves to make changes and get reminders. They will be able to talk to other parents in online forums. The toolkit will let parents know when to seek more help e.g. from their GP.<br><br>**How will the public and patients be involved?**<br>As well as the PaG team, we will have a public and patient involvement (PPI) advisory group. The PPI advisory group will be both experts and parents. They will ensure that the project is relevant, respectful, inclusive, and accessible.<br><br>**What happens next?**<br>We will tell other researchers and the general public about our project results. We will write social media posts, and articles in journals. We will talk at research conferences,

	and do webinars. We will apply for more funding to finish the toolkit, test it with parents and further improve it. We hope the toolkit will be used by the NHS to improve the lives of autistic children and their families.

Our purpose for collecting this data is to communicate with you about your application and have the necessary information to evaluate you for a grant. The data we collect here is collected in the public interest. Information provided here may be subject to Freedom of Information requests.

The NIHR Academy is part of the Department for Health and Social Care (DHSC), National Institute for Health Research (NIHR). The contracting agent for NIHR Academy is the Leeds Teaching Hospital Trust (LTHT). The DHSC is the Data Controller and LTHT is the Data Processor under the General Data Protection Regulation (GDPR) EC 2016/679. DHSC NIHR respects the privacy of individuals who share their data and processes it in a manner that meets the requirements of GDPR. The DHSC Data Protection Officer can be contacted by email at: data_protection@dhsc.gov.uk

The NIHR privacy policy includes further information including ways we may use your data, our contact details and details on your individual rights regarding how your data is used. Your data may be shared across the NIHR, including with other coordinating centres, to allow the application to be managed and for statistical analysis, and with external grant reviewers as part of the process for managing the allocation of a grant. This notice is under constant review and will be updated and / or revised based on that review as appropriate.

Table Of Contents

1. Application Summary Information
2. Applicant CV
3. Applicant Research Background
4. Plain English Summary of Research
5. Scientific Abstract
6. Detailed Research Plan
7. Patient & Public Involvement
8. Training & Development and Research Support
9. Detailed Budget
10. Management & Governance
11. Uploads
12. Participants and Signatories
13. Acknowledgement, Review and Submit

4. Plain English Summary of Research

What is autism and what are feeding problems?
Autism is a condition affecting how people see the world and how they behave. Feeding problems are problems with eating such as only eating a few kinds of foods.

What are the aims of this project?
To develop a toolkit. It will help parents/carers help their autistic children with their feeding problems.

Why is the project important?
Many autistic children have feeding problems. Feeding problems are stressful for them and their families. They can lead to a poor diet. Poor diets can affect a child's health and learning. They can lead to mental and physical health problems, or make existing problems worse. Poor diets are one reason autistic people often die earlier than non-autistic people. Research suggests that parents are often unsure when or if they should ask for help. When they do ask for help, they may find that there is no help for them from their local services. A toolkit could help. It could provide the right support for the right people anywhere and anytime.

What are we going to do?
We will develop a detailed written plan of what we want the toolkit to include. This toolkit will need to meet the needs of children and parents with many different issues. It is a 'complex intervention'.

A team will help with each step of the research, called the *Participatory Research (PaG) team*. It will consist of three each of:

- autistic young people and adults (*experts*)
- parents of autistic children (*parents*)
- professionals who help autistic children with feeding problems (*professionals*)

We will follow established steps to develop a complex intervention:

- **Step 1:** We will search journals and guidelines to see what others think helps with this issue, and why.

- **Step 2:** We will ask other people what they think helps with this issue. We will ask about 50 *experts*, *parents*, and *professionals*. We will draw diagrams to show what helps autistic children with feeding problems and why.

- **Step 3:** We will compare the diagrams from step 2 to *behaviour change theories*. Behaviour change theories explain what helps people change. From them, we will have ideas for what will work in the toolkit. We will write a detailed plan of the contents of the toolkit.

What will the tookit be like?
We imagine the toolkit will be available on a website or app. Parents will answer questions about common problems like sensory issues, or anxiety. They will watch videos and read about things that might help their child. What they see will depend on the answers they gave about their own child. Parents will write goals for themselves to make changes and get reminders. They will be able to talk to other parents in online forums. The toolkit will let parents know when to seek more help e.g. from their GP.

How will the public and patients be involved?
As well as the PaG team, we will have a public and patient involvement (PPI) advisory group. The PPI advisory group will be both experts and parents. They will ensure that the project is relevant, respectful, inclusive, and accessible.

What happens next?
We will tell other researchers and the general public about our project results. We will write social media posts, and articles in journals. We will talk at research conferences, and do webinars. We will apply for more funding to finish the toolkit, test it with parents and further improve it. We hope the toolkit will be used by the NHS to improve the lives of autistic children and their families.

5. Scientific Abstract

Background
Autism is a common complex condition affecting social communication. Many autistic children have feeding problems, typically eating a limited range of foods. Feeding problems affect quality of life, health and development. They lead to lifelong poor diets, which contribute to the 18-year shortened average life expectancy of autistic adults.

Research suggests that parents are often unsure when to or whether to seek help. When they do, local provision of help is often lacking. A toolkit could offer a tailored, multi-component, early intervention to support parents. It would be accessible and scalable.

Research question

Interventions for feeding problems in autistic children. What works, for whom, when, and why?

Aim
To develop an online tool to help parents/carers manage feeding problems in their autistic children.

Objectives
1. To conduct a realist review and construct programme theory(s)
2. To conduct a realist evaluation via interviews of stakeholders and refine the programme theory(s)
3. To co-create a blueprint of a toolkit

Methods
Medical Research Council guidance on developing complex interventions will be followed via three work products (WP):

- **Realist review:** literature search and analysis using realist theory of logic to construct programme theory(s)

- **Realist evaluation:** interviews of stakeholders (autistic young people and adults (*experts*), parents of autistic children (*parents*), and professionals who help parents manage feeding problems (*professionals*)), analysis of verbatim transcripts using realist theory of logic to refine programme theory(s)

- **Co-creation of the toolkit blueprint:** behaviour change theory applied to the programme theory(s) will generate candidate components for the online tool. A blueprint (a detailed textual outline) will be co-created.

A Participatory Research team of experts, parents and professionals will be involved in each WP. Consensus will be reached by asynchronous Nominal Group Technique. An advisory group of experts and parents will ensure that the project is relevant, respectful and accessible.

Timelines for delivery
- Realist review - month 12
- Realist evaluation and programme theory(s) - month 20
- Blueprint - month 26

Anticipated impact and dissemination
Findings of each step will be disseminated via journal publications, conferences and social media.

Further funding will be sought to develop, test and refine a prototype, conduct economic analyses, then finalise a toolkit ready for NHS roll-out. The toolkit will likely be delivered via a website or app. Parents will view content (videos, written content, short courses) tailored to scores on interactive questionnaires, then set goals, get rewards for reaching them, and access peer support via discussion forums. Tailored signposting to other help eg GP will be provided where necessary.

The toolkit would fill a gap in early support for autistic children and families. It could generate big data and enable factional testing of new interventions. It could improve quality of life and diet quality, and reduce longer-term

morbidity and mortality on a large scale.

6. Detailed Research Plan

1. What is the problem being addressed?
Feeding problems in autistic children are common, stressful, and damaging to children's health and development. Feeding problems are "eating-related behaviours that are unrelated to weight, shape, and/or body image concerns, yet impair functioning" (2). Autism is a condition characterised by differences in social interaction and behaviour (3). Feeding problems are five times more common in autistic children compared to non-autistic children and often lead to narrow food repertoires (4).

NB) This proposal adopts the autistic community's preferred terminology: autism (not autistic spectrum disorder) and autistic people (not people with autism) (5,6). Parents is used throughout as shorthand for parents/carers.

Reasons for high rates of feeding problems in autistic people are poorly understood (7). Eating is an unpredictable sensory-rich experience intertwined with complex social rituals; however, combinations of autistic preferences for sameness, social difficulties, and common sensory processing difficulties likely make the eating experience stressful rather than pleasurable (8).

Feeding problems impact a child's and their family's physical and mental health (9). Autistic children are more likely to have micronutrient deficiencies; sometimes with life-threatening or life-changing consequences e.g. vision loss (10–12). Obesity rates are higher in autistic children from as early as two years old (13) and feeding problems contribute to this (14,15). In interviews, parents of autistic children described mealtimes as "one of the most stressful times of the day" (16). Feeding difficulties in autistic children usually persist with advancing age (17).

Surveys have shown that autistic people receive worse healthcare than non-autistic people (18). Many parents are unsuccessful in getting NHS support for diet and nutrition issues, and when they do they don't find the support offered helpful (19). Previous research conducted by the applicant with mothers of autistic children in South London found they were unsure when to or how to seek help with feeding problems, and help was lacking when requested (20).

2. Why is this research important in terms of improving the health and/or wellbeing of the public and/or service users and health and social care services?
Diet is central to health. A high-quality diet prevents early death and disability (21) and improves mental health (22–26). Dietary patterns and preferences laid down in early life often persist to adulthood (27) and therefore targeting feeding problems in childhood promotes lifelong healthier diets. Dietitians are health care professionals skilled in combining knowledge of medicine, nutrition, and dietary manipulation with communication and behaviour change techniques to improve lives.

Autistic adults die on average 18 years younger than the general population and are 2.6 times more likely to die of any cause (28). They have higher rates of nutrition deficiencies, and nutrition-related disorders such as dyslipidaemia, hypertension, diabetes and obesity (29). Mental health conditions are more prevalent (30) and suicide rates are three times higher than in non-autistic people (31, 32).

Addressing feeding problems in autism could reap many rewards; an improvement in nutritional status could improve learning, behaviour, mental and physical health, improve life expectancy, and reduce reliance on social care. It could reduce parental stress, promoting their physical and mental health too. Numerous distressing and costly impacts of feeding problems may be prevented by an early, accessible intervention: hospital admissions due to malnutrition, tube-feeding, intensive feeding therapy, and supports needed after the irreversible damage of micronutrient deficiencies (e.g. vision-loss) (33–35). **This research aims to develop a toolkit to help parents manage feeding problems in autistic children—which will promote lifelong mental and physical health through healthier diets.**

Why a toolkit? And when and how would it be used?
A toolkit would contain various tools—tips or strategies—that could be selected for an individual child's needs. It would be part of the first-line provision of a stepped-care model—with the most effective yet least resource-intensive treatment delivered first; only 'stepping up' to specialist services as clinically required. In other words, this would be a toolkit that would run aside help from health visitors and GPs before referrals are made to dietitians and other specialists. This toolkit would be expected to be made available to families on diagnosis to help the early identification and management of feeding problems, to likely improve diet and quality of life for many families, but some would still need individualised specialist help.

The best **mode of delivery** of the toolkit is one of the things the research will determine. It is likely at least some of the toolkit will be online. An online toolkit holds many potential benefits: most parents in the applicant's previous research expressed that they would welcome tailored online help due to difficulty attending appointments (20). Trials of digital health interventions (DHIs) for parents of autistic children have established efficacy, with engagement and outcomes being comparable to face-to-face interventions (36). DHIs are cost-effective, easily accessible to those who find it difficult to attend face-to-face appointments (37), and can help reduce parents' stress and help them stay at work (38). Additionally, an online tool offers the potential for an easily scalable provision that if rolled out nationally could generate big data to drive a better understanding of feeding problems and test new approaches, even further improving the lives of autistic children and their families. However, the toolkit must be accessible. Although the vast majority of the target market for the toolkit are 'internet users' i.e. have the skills and equipment to access the internet, up to 3% of 18-55-year-olds aren't regular internet users, and there are differences in internet usage between ethnic groups (notably less in Bangladeshi adults) and regions (lowest in the North East of England) (39) Having a toolkit available in both online and paper formats may be necessary to ensure accessibility for all.

Public and Patient Involvement (PPI) input when developing this proposal was universally positive that the proposed toolkit would be very useful:

- Contributor 3, an autistic adult, said "t*his project is very relevant […] As there is so little understanding and support of eating difficulties in (autistic) adults also, this can then mean a lifelong struggle. It is best to be addressed as early as possible*".

- Contributor 2, a non-autistic mother of an autistic child said, "*Being able to access more support via online websites would be beneficial. Past experiences have been long awaited appointments, with no follow-ups or too long between each appointment"*.

- Contributor 1, an autistic mother of an autistic child said, *"I think that the project is interesting in that it encourages personal agency and advocacy as well as self-care and autonomy and viewing food in a positive way*".

Reducing health inequalities for autistic people is a national priority: of Public Health England, the NHS Long Term Plan, and the National Autism Team (40, 41). Improving diets is a key part of that. NICE recommends that autistic children should be assessed for feeding problems and restricted diets and referred on, however to whom is not specified (3).

A top-ten priority for autism research identified by a **James Lind Alliance (JLA) Priority Setting Partnership** was "How can parents and family members be supported/educated to care for and better understand an autistic relative" (42). This is a fundamental aim of this research. The project also fits eleven other JLA autism priorities including those related to mental health, sensory processing, challenging behaviour, early interventions and a better understanding of what it is like to be autistic.

This research is innovative and is relevant to patients and the public; it is aligned with the **National Institute for Health Research Clinical Research Network (NIHR CRN) allied health professionals (AHPs) strategy goals** 3: support AHPs to deliver research that is relevant to patients and public and 4: support AHPs to embrace innovation in their research roles (43).

This will be the first toolkit developed for this purpose.

3. Review of existing evidence - How does the existing literature support this proposal?
A scoping review was carried out in April 2021 for interventions for feeding problems in autistic children using subject headings and keyword terms in several databases. Following screening, 21 relevant records were found: 3 systematic reviews (SR), and 18 empirical studies. Case studies and case series were excluded. An additional search of trial registry databases found one related ongoing trial.

Two 2019 SRs focussed on feeding problems in children and adults with developmental disorders that included autism. Both reviewed interventions for increasing the acceptance of new foods. **Both concluded that there is a lack of evidence for interventions to improve dietary intake in children and adults with developmental disorders** (44, 45).

The third SR conducted a meta-analysis of interventions to improve feeding difficulties in autistic children retrieved up to 2013. 23 low-quality studies were identified, all with fewer than five participants. All used applied behavioural

analysis (ABA) interventions (a controversial intensive and invasive approach not typically used in the UK). **The conclusion was that further research was needed (46).**

The 18 empirical studies identified were all published since the third SR:

- Six evaluations of approaches each involved fewer than five patients but showed promising results: two occupational therapy-led (47, 48); one dietitian-led (50); one speech-and-language therapist-led (51); and two weight-management clinics tailored for autistic children (52, 53).
- Six pilot studies of child and family group education sessions reported high adherence rates, high parent satisfaction, and promising improvements in dietary quality (54–59).
- Three of four pilot studies of school-based interventions with children showed promise (60–63); two interventions that had not yet been piloted were also described (64, 65).
- One ongoing pilot study of a home-based intervention was retrieved from trial registries (66).

These studies demonstrate that various approaches exist and that multiple disciplines have useful approaches that could be incorporated into the proposed toolkit e.g. parental education and sensory exposure.

The Medical Research Council define a complex intervention as one that has complexities such as interactions between components; number and difficulty of behaviours; variability of outcomes and tailoring of intervention (67). In a survey study of autistic (n=56) and non-autistic (n=172) children with feeding problems and children with no feeding problems (n=259), feeding problems were shown to be worse in autistic children but have similar underpinnings including behavioural problems and sensory hypersensitivity (68). **This study further supports the need for complex intervention development to help with feeding problems in autistic children.**

SRs of interventions to improve feeding problems for non-autistic children also find high-quality evidence elusive (69, 70). However, an SR of digital health interventions (DHIs) to improve healthy eating in children and adolescents found statistically significant positive changes in eating behaviour (15 studies) (71), and an SR of DHIs to change parental feeding practices found promising improvements too (12 studies) (72). Given the overlap in underpinnings in feeding problems in autistic and non-autistic children, these SRs further demonstrate that **DHIs are a promising avenue for helping with feeding problems and improving dietary intake in autistic children.**

A mixed-methods NIHR-funded study: FEEDS, published in March 2021 incorporated systematic review updates, and evidence syntheses with surveys, interviews and workshops with the aim to examine interventions to be delivered at home by parents to improve eating, drinking and swallowing in young children with neuro-disability (including autism) (73). They found limitations in available research evidence. They conducted a survey of 359 parents, 421 health professionals (HPs), and 62 education professionals. 51% of the parents had an autistic child. They found that parents and professionals reported using multiple and differing interventions tailored to need, many perceived as effective. They synthesised their results from their SR, survey and focus groups. An online Delphi process of 196 parents and 175 HPs identified 19 essential interventions (including parent support and many that could be delivered for parents via a toolkit) and 10 outcomes (including nutrition, quality of life for the child and mental health for the parent). The research concluded that there is a need for further research, a disparity of service provision nationally to help children with feeding problems and particularly a lack of any support for autistic children. **They found a need for 'a flexible toolkit of interventions'**, they recommended that ideally, health professionals should deliver the toolkit alongside parents however to reach those poor service access **"innovative technology-based solutions should be considered".** They conclude that **future research funding should be provided for complex intervention development.**

4. What is the research question / aims and objectives?
Research question:

- Interventions for feeding problems in autistic children. What works, for whom, under what circumstances, and why?

Aim

- To develop a toolkit that helps parents/carers manage feeding problems in autistic children; to improve their short term and long term health, development and quality of life.

Objectives

- To conduct a realist review to identify important components for the tool and to understand the mechanism, context and outcome of successful interventions to result in the co-creation of programme theory(s)
- To conduct a realist evaluation via interviews of stakeholders to test, refine and finalise the programme theory (s)
- To co-create a blueprint of a toolkit, based on the realist review and evaluation

5. Project Plan

The tool development will follow the MRC guidance for complex intervention development (67) and will involve a Participatory Research team.

Participatory Research team

Patient and public involvement in intervention development ensures that it truly benefits those it is being designed for. As well as the PPI advisory group, a Participatory Research (PaR) team will be closely involved in this research. Figure 1 in uploads illustrates the different roles of the PaR, PPI and steering groups throughout the research.

The PaR team will consist of three autistic young people and adults who have had feeding problems (*experts*), three parents of autistic children who have or have had feeding problems (*parents*), and three professionals who help autistic children and their families with feeding problems (*professionals*). Autistic young people and adults are included throughout this research as experts on the lived experience (74, 75). Younger people will be included as they will have more recent relevant experience of being a child, to add their important insights.

The team will be recruited purposively via social media, Autistica and/or personal contacts and involved in partnership with the primary researcher at key parts of the research process.

Research methods training for the PaR team will be provided by the primary researcher and core supervisory team throughout.

All PaR team activity will be carried out with accessibility and inclusivity at the forefront, in line with Academic-Autistic Spectrum Partnership in Research and Education (AASPIRE) guidelines for the inclusion of autistic adults in research as co-researchers and study participants, examples of reasonable adjustments are as detailed in the PPI section (76).

An asynchronous nominal group technique (NGT) (77, 78) will be used to obtain consensus from the PaR team at each step. Asynchronous NGT ensures accessibility for those who have slow processing speeds, need to use assisted communication, or who may find face-to-face/online synchronous group discussions and more complex consensus techniques challenging. It also reduces power differentials that can skew a consensus process when being carried out face-to-face where for example professional voices may dominate (76, 77).

The stages to the NGT are:

- Options that need to be chosen are presented to the PaR team via video and/or text.
- Team members individually comment on the content using an online collaborative tool (Padlet).
- Team members group content on the importance of inclusion.
- Primary researcher re-presents findings.
- Team members rate each piece of previously selected content.
- The process continues until a final agreement has been reached (76, 77)

Work packages

The toolkit is a complex intervention: the Medical Research Council (MRC) framework for complex interventions sets out that the development stage includes: 1. Identifying the evidence base; 2. Identifying/developing theory; and 3. Modelling process and outcomes (67, 79, 80). The timeline for the research is depicted in a Gantt chart in the upload section. Each of these will be completed via the incremental work packages (WP) below:

WP1: a realist review
Aims

- To identify the evidence base and develop programme theory(s)

Objectives

1. To conduct a realist review to identify the mechanism, context and outcome of successful interventions.
2. To develop programme theory(s)

Methods
Complex interventions must have a coherent theoretical basis to be an intervention worth implementing and to ensure the best return on investment (67). A systematic review was initially considered for this first step. However, as the scoping review has confirmed that the evidence in this field is limited, a realist review will be carried out. Realist approaches aim to what works, for whom and in what circumstances and why. A realist review will therefore generate more transferable findings than systematic reviews (81).

The steps below are incremental and iterative. They are in line with RAMESES (Realist And Meta-narrative Evidence Syntheses: Evolving Standards) (82) and the successful NIHR-funded realist synthesis carried out by collaborator and advisor on this project Dr Ian Maidment, related to medicine management in older people (MEMORABLE) (49, 83).

Steps:
1. Review registration: with PROSPERO (the NIHR International prospective register of systematic reviews)

2. Scoping search: Initial theories will be identified via broad searches in MEDLINE, SCOPUS and CINAHL, related to the research question. Relevant systematic reviews and guidelines will be retrieved and theory–rich papers identified from their bibliographies.

3. Theory generation: In conjunction with the PaR team, initial programme theory(s) will be generated from records retrieved in the scoping search. Programme theory sets out how and why outcomes occur within an intervention and is often depicted as a visual logic model (84). In this step, the initial theory is a 'rough and ready' depiction in order to inform the next step.

4. Systematic search: A search strategy will be developed iteratively from steps 2 and 3 using the CIMO (Context, Intervention, Mechanisms, Outcome) question framework, to continue to construct a plausible, coherent programme theory. Searches will include academic databases (MEDLINE, SCOPUS and CINAHL) and grey literature (reports, toolkits, policy documents). Reference lists of relevant documents will be checked for additional material.

5. Screening and sifting: Titles and abstracts, then full text will be screened for inclusion based on relevance and rigour. Rigour here differs from a traditional systematic review as realist reviews reject the usual hierarchy of evidence quality but instead looks for evidence that is trustworthy and theory-rich enough to be useful to the research question (81).

6. Data extraction: Full text of included records will be imported into NVivo (QSR International) for coding deductively and inductively. Codes will cover concepts that are important and potentially relevant to the programme theory(s): data will be coded as context, mechanism or outcome.

7. Data analysis and synthesis: In conjunction with the supervisory and PaR team the programme theory(s) will be refined. Consensus will be reached via NGT. Programme theory will be generated using a realist logic of analysis, the building of context-mechanism-outcome configurations (CMOC) that describe how contextual factors (C) trigger particular mechanisms (M) to generate various outcomes (O) and therefore to understand how an aspect of an intervention works or might be expected to work in what conditions (85).

WP2: a realist evaluation
Aims
- To test and refine the programme theory(s)

Objectives
- To conduct interviews with experts, parents, professionals to test and refine the programme theory(s)

Methods:
Following the methodology used in the MEMORABLE study (83), realist-informed interviews will be carried out:

Steps:
1. Interview schedules: With the PaR team, informed by WP1 findings, realist-informed interview schedules

will be created to explore the research question. Interviews will be different for each stakeholder group: experts, parents, and professionals. Interview schedule content consensus will be reached via NGT. Piloting of the interview schedules and accessibility checks will be carried out with the PPI group.

2. Recruitment: purposive: experts, parents and professionals via adverts through Autistica Insight Network and social media; commissioners via social media and snowball sampling. Care will be taken to involve participants with intersectionality of characteristics proffering higher risk of health inequalities e.g. those with intellectual disabilities and from Black, Asian and Minority Ethnic (BAME) communities.

   Targeted inclusion of autistic people and their families from specific BAME groups is key to ensuring the tool captures factors useful for these groups. Differing feeding practices between ethnic groups from infancy result in different growth patterns predictive of different health outcomes (86–88). Black, Pakistani and Bangladeshi adults experience higher rates of diet-related health inequalities that are not explained by other factors such as deprivation (89). BAME autistic people and their families are underrepresented in research and often find services less accessible to them due to a lack of cultural competence (90, 91).

3. Interviews: will be held online via a mode and time chosen by participants as best for them, e.g. Microsoft (MS) Teams video, phone, text chat, face-to-face with assisted communication (if local). Participant information and consent forms will be provided to participants before the groups or interviews in accessible formats. Demographic information will be collected to evaluate the representativeness of the sample related to the identified equality, diversity and inclusivity aspects. Costing for interpreters and expert consultants to advise and assist to enable full participation for those using assisted technology has been included (e.g. speech and language therapists or autism education specialists). Questions will be given in advance of the interviews to enable those with processing differences or using assisted communication to formulate responses. Interviews will be recorded (by MS Teams/digital recorder as appropriate) and transcribed verbatim. Interviews will follow the schedule but be flexible to allow exploration of causal accounts as they emerge. They will take approximately an hour.

   Sampling: Data will be analysed in batches after five interviews of each group are carried out and then continued until adequate depth and breadth of data has been collected (saturation achieved). It is estimated this will be 15-30 interviews in each group.

4. Analysis: Transcriptions will be coded in NVivo using the same realist logic of analysis as WP1. The interviewee data will be analysed and coded for the underpinning CMOC.

5. Synthesis: With the supervisory and PaR team, the CMOC's from WP1 and WP2 will be synthesised, looking for consistent patterns and emerging programme theory(s). Findings will be discussed and further iterations of analysis undertaken as necessary. Final programme theory(s) will be co-produced and consensus reached via NGT.

WP3: tool co-design
Aim:
- To co-create a blueprint of the toolkit from the programme theory(s) generated in WP1 and WP2

Objective:
- To co-create a process model and outcomes of a toolkit by applying behaviour change theory to the programme theory
- To co-design a blueprint of the toolkit from the emergent behaviour change candidates

Methods:
The programme theory(s) developed in WP1 and 2 will be used in this final stage of development to model the process and outcomes of the toolkit and a subsequent blueprint (outline).

Steps
1. **Process and outcome modelling:** In conjunction with the supervisory and PaR teams, a 'big theory' (e.g. behaviour change theory) will be applied to the emergent programme theory(s). The big theory(s) chosen will depend on the findings: as the key principle of complex intervention design is flexibility and iteration (67).

   A strong case has been made for the inclusion of behaviour change theory into complex intervention development (79, 92). Decades of cross-discipline applied research has enabled the rigorous

characterisation of the 'active ingredients' of interventions such as goal setting, graded tasks, instruction, and social supports into the Behaviour Change Techniques Taxonomy (v1) (BCTTv1) (93). 93 distinct Behaviour Change Techniques (BCTs) have been identified and mapped via expert consensus to theoretical constructs underlying behaviour change e.g. knowledge, goals, optimism (the Theoretical Domains Framework (TDF)) (94). Having a common international language in which to describe behaviour change components of interventions is key for comparison, replication, and trials of successful components and combinations of components.

By mapping the programme theory(s) to the TDF, candidate BCTs can be identified from the BCTTv1 which would be likely to facilitate change in the behaviours that will be needed to overcome feeding problems in autistic children. From these candidate BCTs, the co-design group will select both the BCTs to be included and the mode of delivery for each BCT.

2. **Blueprint co-design:** Using the process and outcome modelling, a blueprint (a detailed outline e.g. textual descriptions and diagrams on a Word document) will be co-designed. This will involve the PaG, supervisory teams and a web-developer or digital health intervention expert (as a digital element is likely). The NGT consensus process will be used, with iterative rounds until a finished product has been co-designed.

The content and delivery of the toolkit will be decided following the stages in this project. It is envisaged that the toolkit will be best delivered either in its entirety or in conjunction with a website/app that is behind a log-in wall. The parent would complete questionnaires (e.g. validated nutrition and feeding screening tools) and then access content tailored to their answers. This content will be incorporated from existing sources or created for the tool, and take the form of videos, written content, and short training courses. Other components of the toolkit may be forums, 'ask the expert' sections, or gamification (badges, rewards). Tailored signposting to other services e.g. GP, will be given when deemed necessary by scores on questionnaires.

The blueprint will be ready for further development after the PhD programme either via commercial, charity or research funding.

6. Dissemination, Outputs and Anticipated Impact
Dissemination
The research findings will be disseminated via:
1. Research publications: Project protocol; realist review and protocol; realist evaluation; final programme theory(s); PhD thesis.
2. Lay publications: articles for blogs and National Autistic Society magazines
3. Conference abstracts/posters for each work package
4. PPI group dissemination via Autistica and other networks
5. Social media channels, blogs, podcasts and webinars
6. End of project online dissemination event - invited speakers and PPI group disseminating findings

Outputs
The primary output of the research will be a blueprint for a toolkit to help parents manage feeding problems in autistic children.

Secondary outputs will be the research publications as detailed in the dissemination section.

Anticipated impact
This project will increase our understanding of feeding problems in autistic children and be the first to engage autistic young people and adults as participants as experts by experience.

We will seek further funding to finalise the toolkit as per the MRC framework: prototype production, feasibility testing, refining the toolkit; multiphase optimization strategy (MOST) testing of included elements to determine the most effective combination (95), trial and health economic analysis of the final toolkit and, if favourable, rollout to NHS trusts.

If shown to be effective in future testing, the finalised toolkit will have a far-reaching impact:
- For autistic children: improved diet quality which will impact learning, development, mental and physical health, quality of life, and potentially lengthen life expectancy.
- For families of autistic children: better supported, improved quality of life.
- For health professionals: access to a quality first-line tool to refer parents to for feeding problems (as per NICE guidance); time freed up from providing first-line support.
- For commissioners and service providers: access to a cost-effective scalable and trustworthy tool.

The toolkit will raise awareness of feeding problems in autistic children and gaps in NHS services, driving service improvement.

The health economic benefits of improving the health and capacity to learn for autistic children and improving the health of their families could be significant.

Additionally and crucially this toolkit will offer the potential for anonymised data gathering to better understand the dietary quality, and eating behaviours of autistic children in England on a larger scale than has previously been possible, analysis of which could inform new theories (e.g. through machine learning), interventions and gaps in provisions and generate new research avenues, plus the potential to trial other novel intervention packages via the platform.

7. Project Management
I will have overall responsibility for running the day-to-day project.

A Steering Group will meet quarterly to support me to critically evaluate progress, advise on logistical issues including risk, financial and intellectual property management, and ensure the project has maximum impact. This group will include at least one representative from the PPI group, Aston University, and UHCW NHS Trust. Figure 1, a schematic comparing the roles of the steering group with the PPI group and the PaR group is in uploads in Appendix 2.

I will have contact with my primary supervisor weekly, and my additional supervisors at least quarterly.

UHCW NHS Trust and Aston University Finance Teams will ensure adherence to financial management. The two sites will manage the costs incurred at either site.

A Gantt chart outlining the timeline of the project is in uploads as Appendix 3.

8. Ethics
This project will be carried out in accordance with the Helsinki declaration, the Health and Care Professions Council Code of Conduct and NIHR and local ethical guidelines (96–99). Ethical considerations are:
- Realist review: no primary data collected therefore no ethical issues nor approval needed

- Realist evaluation and Participant Research team: Data collection involves the general public and includes young people and vulnerable adults plus various professionals. Ethics committee approval will be via Aston University and IRAS. Production of participant information, collection of informed consent and appropriate debriefing will be carried out in line with NIHR and AASPIRE best practice guidance, and PPI co-created (76, 98). All sensitive data collected for this study will be treated confidentially and stored securely in accordance with the Data Protection Act 2018 (100). Due to the potential inclusion of under-16s, assent would be sought from them plus additional consent from parents/carers and care taken to ensure online safety (101).

9. Success Criteria
1. Completion of realist review and initial programme theory(s) by month 12

2. Completion of realist evaluation and final programme theory(s) by month 20

3. Completion of blueprint by month 26

Risks to research delivery
1. Delayed milestones – mitigated by: clear project management plan, mentoring from supervisors and collaborators, support from the clinical research network, steering group meetings

2. Poor recruitment of participants – mitigated by: advertising widely via existing networks known to the research team and collaborators, PPI representatives, methods suggested by PPI representatives

Risks to the successful completion of this project and delivery of the blueprint are very low.

7. Patient & Public Involvement

Please describe how patients/service users, carers and the public have been involved in developing this proposal

This project idea came from discussions with parents and professionals over many years in the applicant's clinical practice highlighting the significant gaps in provision by the NHS and bolstered by the findings from the applicant's qualitative study of parents of autistic children with feeding problems (20).

Contributors
PPI contributors to this application were recruited via Autistica's Insight group and asked for specific feedback via email on the project idea, plain English summary, PPI involvement, inclusivity, and accessibility. Three of four invited contributors responded. All were reimbursed for their involvement as per INVOLVE guidance (102) using the West Midlands NIHR Research Design Service Public Involvement Fund.

- Contributor 1 is a 47-year-old autistic mother to a 15-year-old autistic boy, both have feeding problems
- Contributor 2 is a non-binary 28-year-old autistic person who has had feeding problems
- Contributor 3 is a non-autistic mother to a 14-year-old autistic boy who has feeding problems.

Positive feedback:

- All thought that the project was useful and relevant, and the title clear.
- Contributors 1 and 3 were happy with the plain English summary.
- Things they liked about the project included:
    - Contributor 2: "I like that autistic people, and their families, are being approached and involved in this project. **I like that a significant, and life-altering, difficulty is being explored and addressed.**"
    - Contributor 3: "Support being available via online website"
    - Contributor 2 said of the inclusivity elements: "I think it is an excellent approach. I like that autistic people and families are being consulted, and that care is taken to create a respectful, sensitive, and supportive environment for participation."

Changes made in response to PPI feedback:

- Changes suggested by contributor 2 to the plain English summary
- Improvements suggested to the accessibility and inclusivity of participants and contributors, which included: to schedule regular breaks; to be sent a clear agenda/ interview questions beforehand; giving participants choices when to do interviews; allowing participants to change their method of communication at any point; ensuring any team building activities are optional and informal; acknowledging PPI members on any outputs. All contributors expressed interest in being on the PPI advisory board of this project.

Please describe the ways in which patients/service users, carers and the public will be actively involved in the proposed research, including any training and support provided

A 6-person PPI advisory group will ensure the research is relevant, respectful, and inclusive, throughout the project. The board will include parents of autistic children, and autistic young people and adults.

Recruitment will be via Autistica Insight, social media, and support groups, with specific calls for autistic people using different communication modes, autistic people with intellectual disability, and autistic people and/or parents from BAME communities.

AASPIRE guidelines will be used to ensure that meetings are fully accessible and to address power differences (76) including:

- Meetings carried out using Microsoft Teams video with the option for asynchronous participation for anyone unable to attend due to work/school or illness

- Agenda circulated in advance to allow time for any contributors who need processing time or to prepare responses using assisted communication

- Use of a live transcriber to ensure that any contributors with auditory processing or hearing impairment will have accurate subtitles. (The costs for the use of a live transcriber and included in the finances. Whilst this is a significant cost, addressing accessibility and power in these meetings is critical to their success)

The group will meet three times a year for two hours. Initial meetings will involve non-threatening team-building activities and there will be a social aspect to each meeting to promote commitment to continued contribution. Training in research methods will be carried out when necessary.

Contributions will include:

- Advising on the relevance, respectfulness, and inclusivity of:
    - the project plan
    - ethics proposals
    - interview questions
    - participant information sheets
    - consent forms
    - recruitment
    - dissemination materials and activities
    - emerging findings

- Assisting with:
    - Recruitment of participants
    - Reporting (contributing to the reporting layout and design and content)
    - Dissemination (finding audiences, designing infographics and videos). All contributors will be acknowledged in dissemination materials if they wish to be.

All PPI involvement will be as per NIHR, local and professional codes of conduct (96-99, 103). Care will be taken to ensure online safety for all participants but particularly included children e.g. contact details will not be shared to prevent any direct contact between contributors. All contributors will be reimbursed for their involvement as per INVOLVE guidance.

**If it is considered not appropriate and meaningful to actively involve patients/service users, carers and the public in your proposed research, please justify why**
not applicable

Appendix 1: References

1. D. Baltruks, P. Callaghan, "Nursing, midwifery and allied health clinical academic research careers in the UK" (2018), (available at www.councilofdeans.org.uk,@councilofdeans).
2. J. Baraskewich, K. M. von Ranson, A. McCrimmon, C. A. McMorris, *Autism*, 136236132199563 (2021).
3. NICE: National Institute for Health and Care Excellence, "Guideline CG128: Autism in under 19s: recognition, referral and diagnosis" (NICE, London, UK, 2011).
4. K. M. Peterson, C. C. Piazza, V. F. Ibañez, W. W. Fisher, *Journal of applied behavior analysis*. **52**, 895–917 (2019).
5. L. Kenny *et al.*, *Autism*. **20**, 442–462 (2016).
6. National Autistic Society, How to talk about autism, (available at https://www.autism.org.uk/what-we-do/help-and-support/how-to-talk-about-autism).
7. J. Baraskewich, K. M. von Ranson, A. McCrimmon, C. A. McMorris, *Autism* (2021), doi:10.1177/1362361321995631.
8. T. M. Dovey, V. Kumari, J. Blissett, *European Psychiatry*. **61**, 56–62 (2019).
9. W. G. Sharp *et al.*, *Journal of autism and developmental disorders*. **43**, 2159–73 (2013).
10. S. Yule *et al.*, *Journal of the Academy of Nutrition and Dietetics* (2020), doi:10.1016/j.jand.2020.10.017.
11. W. G. Sharp, R. C. Berry, L. Burrell, L. Scahill, B. O. McElhanon, *Journal of developmental and behavioral pediatrics : JDBP*. **41**, 397–405 (2020).
12. M. H. Zimmer *et al.*, *Journal of Autism and Developmental Disorders*. **42**, 549–556 (2012).
13. A. P. Hill, K. E. Zuckerman, E. Fombonne, *Pediatrics*. **136**, 1051–1061 (2015).
14. K. K. Dhaliwal, C. E. Orsso, C. Richard, A. M. Haqq, L. Zwaigenbaum, *International Journal of Molecular Sciences*. **20**, 3285 (2019).
15. M. V. Nadeau, E. Richard, G. L. Wallace, *Journal of Autism and Developmental Disorders* (2021), doi:10.1007/s10803-021-04945-6.
16. K. Marquenie, S. Rodger, K. Mangohig, A. Cronin, *Australian Occupational Therapy Journal*. **58**, 145–54 (2011).
17. S. D. Page, M. C. Souders, T. V. E. Kral, A. M. Chao, J. Pinto-Martin, *Journal of Autism and Developmental Disorders* (2021), doi:10.1007/s10803-021-04947-4.
18. The Westminster Commission on Autism, "A Spectrum of Obstacles: An Inquiry into Access to Healthcare for Autistic People" (London, 2016).
19. J. Wills, Y. Evans, "Health and service provision for people with Autism Spectrum Disorder: A survey of parents in the United Kingdom, 2014" (London, UK, 2016).
20. Z. L. Connor, thesis, Coventry University, Coventry, UK (2017).
21. A. Afshin *et al.*, *The Lancet*. **393**, 1958–1972 (2019).
22. S. Khalid, C. M. Williams, S. A. Reynolds, *British Journal of Nutrition*. **116**, 2097–2108 (2016).
23. R. S. Opie, A. O'Neil, C. Itsiopoulos, F. N. Jacka, *Public Health Nutrition*. **18**, 2074–2093 (2015).
24. J. S. Lai *et al.*, *The American Journal of Clinical Nutrition*. **99**, 181–197 (2014).
25. A. O'Neil *et al.*, *American Journal of Public Health*. **104**, e31–e42 (2014).
26. H. M. Francis *et al.*, *PLOS ONE*. **14**, e0222768 (2019).
27. M. H. Pesch, K. W. Bauer, M. J. Christoph, N. Larson, D. Neumark-Sztainer, *Public Health Nutrition*. **23**, 987–995 (2020).
28. Autistica, "Personal tragedies, public crisis The urgent need for a national response to early death in autism" (2016).
29. L. A. Croen *et al.*, *Autism*. **19**, 814–823 (2015).
30. M. C. Lai *et al.*, *The Lancet Psychiatry*. **6**, 819–829 (2019).
31. K. Kõlves, C. Fitzgerald, M. Nordentoft, S. J. Wood, A. Erlangsen, *JAMA network open*. **4**, e2033565 (2021).
32. T. Hirvikoski *et al.*, *British Journal of Psychiatry*. **208**, 232–238 (2016).
33. K. E. Williams, *Article in Journal of Developmental and Physical Disabilities* (2007), doi:10.1007/s10882-007-9051-y.
34. M. J. Bruins, J. K. Bird, C. P. Aebischer, M. Eggersdorfer, Considerations for secondary prevention of nutritional deficiencies in high-risk groups in high-income countries. *Nutrients*. **10** (2018), p. 47.
35. R. Dempster, W. Burdo-Hartman, E. Halpin, C. Williams, *Journal of Pediatric Psychology*. **41**, 857–866 (2016).
36. B. Ingersoll, N. I. Berger, *Journal of Medical Internet Research*. **17** (2015), doi:10.2196/jmir.4913.
37. A. McAuley, *Public Health*. **128**, 1118–1120 (2014).
38. Carers UK, "Potential for Change: Transforming public awareness and demand for health and care technology" (London, UK, 2013), , doi:10.1016/0011-684X(85)90054-1.
39. Office for National Statistics, Exploring the UK's digital divide - Office for National Statistics (2019), (available at https://www.ons.gov.uk/peoplepopulationandcommunity/householdcharacteristics/homeinternetandsocialmediausage/articles/exploringtheuksdigitaldivide/2019-03-04).
40. "The NHS Long Term Plan" (2019).
41. NHS England, NHS England » National Autism Team Update, (available at https://www.england.nhs.uk/learning-disabilities/about/national-autism-team-update/).
42. Autistica, James Lind Alliance, "Your questions: shaping future autism research" (2016).
43. National Institute for Health Research (NIHR), "NIHR CRN Allied Health Professionals Strategy 2018-2020" (2018).
44. S. King, H. E. Johnson, T. Burch, A. Chitiyo, *Research and Practice for Persons with Severe Disabilities*. **44**, 169–185 (2019).
45. L. R. Chawner, P. Blundell-Birtill, M. M. Hetherington, *Journal of Autism & Developmental Disorders*. **49**, 3504–3525 (2019).
46. J. Marshall, R. Ware, J. Ziviani, R. J. J. Hill, P. Dodrill, *Child: Care, Health and Development*. **41**, 278–302 (2015).
47. J. Cosbey, D. Muldoon, S. Shelly, K. Ledingham, *American Journal of Occupational Therapy*. **70**, 1 (2016).
48. H. Hillman, *Physical & Occupational Therapy In Pediatrics*. **39**, 629–641 (2019).
49. I. Maidment *et al.*, *Systematic Reviews*. **6** (2017), doi:10.1186/s13643-017-0528-1.
50. K. Maclin, J. Kandiah, A. Haroldson, J. Khubchandani, *Journal of the Academy of Nutrition & Dietetics*. **117**, A29–A29 (2017).
51. D. Muldoon, J. Cosbey, *American Journal of Speech-Language Pathology*. **27**, 278–287 (2018).
52. J. C. Espinoza *et al.*, *Autism: The International Journal of Research & Practice*, 1 (2021).
53. M. L. D. Gillette *et al.*, *Journal of developmental and behavioral pediatrics : JDBP*. **35**, 266–273 (2014).
54. W. G. Sharp *et al.*, *Journal of Pediatrics*. **211**, 185-192.e1 (2019).
55. W. G. Sharp *et al.*, *Journal of Autism and Developmental Disorders*. **43**, 2159–2173 (2013).
56. T. L. Burrell *et al.*, *Seminars in Pediatric Neurology*, in press (available at http://search.ebscohost.com/login.aspx?direct=true&AuthType=ip,sso&db=ccm&AN=145496813&site=ehost-live&authtype=sso&custid=s9872838).
57. C. R. Johnson *et al.*, *Journal of Pediatric Psychology*. **44**, 164–175 (2019).
58. E. S. Kuschner *et al.*, *Clinical Child and Family Psychology Review*. **20**, 403–421 (2017).
59. A. Miyajima *et al.*, *Hong Kong Journal of Occupational Therapy*. **30**, 22–32 (2017).
60. A. Buro, H. Gray, *Journal of Nutrition Education & Behavior*. **52**, S12–S13 (2020).
61. H. J. Cassey, Y. Washio, D. A. Hantula, *Delaware medical journal*. **88**, 342–345 (2016).
62. L. M. Y. Chung, Q. P. S. Law, S. S. M. Fong, *Journal of Alternative & Complementary Medicine*. **26**, 1074–1079 (2020).
63. C. Taylor, P. Upton, D. Upton, *Education & Health*. **31**, 95–97 (2013).
64. S. F. N. Freeman *et al.*, *Beyond Behavior*. **28**, 142–153 (2019).
65. W. van Arsdale, H. Gray, A. Buro, *Journal of Nutrition Education & Behavior*. **52**, S87–S87 (2020).
66. ClinicalTrials.gov Identifier: NCT03071120, Parent-Mediated Intervention for Families With Children With Autism and Feeding Challenges - Full Text View - ClinicalTrials.gov, (available at https://clinicaltrials.gov/ct2/show/NCT03071120?term=feeding&cond=Autism&draw=2&rank=3).
67. P. Craig *et al.*, *BMJ*. **337**, a1655 (2008).
68. T. M. Dovey, V. M. Kumari, J. Blissett, *European Psychiatry*. **61**, 56–62 (2019).
69. M. M. Gosa, H. T. Carden, C. C. Jacks, A. Y. Threadgill, T. C. Sidlovsky, Evidence to support treatment options for children with swallowing and feeding disorders: A systematic review. *Journal of Pediatric Rehabilitation Medicine* (2017), , doi:10.3233/PRM-170436.
70. W. G. Sharp, V. M. Volkert, L. Scahill, C. E. McCracken, B. McElhanon, *Journal of Pediatrics* (2017), doi:10.1016/j.jpeds.2016.10.002.
71. L. M. Hamel, L. B. Robbins, Computer- and web-based interventions to promote healthy eating among children and adolescents: A systematic review. *Journal of Advanced Nursing*. **69** (2013), pp. 16–30.
72. A. I. Gomes, A. I. Pereira, M. S. Roberto, K. Boraska, L. Barros, *PLOS ONE*. **16**, e0250231 (2021).
73. J. Parr *et al.*, *Health Technology Assessment*. **25**, 1–208 (2021).
74. K. Gillespie-Lynch, S. K. Kapp, P. J. Brooks, J. Pickens, B. Schwartzman, *Frontiers in Psychology*. **8** (2017), doi:10.3389/fpsyg.2017.00438.
75. D. E. M. Milton, *Autism*. **18**, 794–802 (2014).
76. C. Nicolaidis *et al.*, *Autism*. **23**, 2007–2019 (2019).
77. K. L. Dowling, R. D. st. Louis, *Decision Support Systems*. **29**, 229–248 (2000).
78. K. Manera, C. S. Hanson, T. Gutman, A. Tong, in *Handbook of Research Methods in Health Social Sciences* (Springer Singapore, Singapore, 2019), pp. 737–750.
79. A. O'Cathain *et al.*, *BMJ Open*. **9**, e029954 (2019).
80. N. Bleijenberg *et al.*, *International Journal of Nursing Studies*. **79**, 86–93 (2018).
81. R. Pawson, T. Greenhalgh, G. Harvey, K. Walshe, Realist review - A new method of systematic review designed for complex policy interventions. *Journal of Health Services Research and Policy*. **10** (2005), pp. 21–34.
82. G. Wong *et al.*, *Health Services and Delivery Research*. **5**, 1–108 (2017).
83. I. Maidment *et al.*, *BMC Geriatrics*. **20** (2020), doi:10.1186/s12877-020-01568-x.
84. P. J. Rogers, (2008), doi:10.1177/1356389007084674.
85. R. Pawson, N. Tilley, *Realistic Evaluation* (Sage, 1997).
86. E. M. Perrin *et al.*, *Pediatrics*. **133**, e857 (2014).
87. O. Sirkka *et al.*, *BMC Pediatrics*. **21** (2021), doi:10.1186/s12887-020-02456-4.
88. C. L. Falconer *et al.*, *BMJ Open*. **4**, 3949 (2014).
89. Race Equality Foundation, "Healthy eating in UK minority ethnic households: Influences and way forward" (London, UK, 2016), (available at https://raceequalityfoundation.org.uk/health-care/healthy-eating-in-uk-minority-ethnic-households-influences-and-way-forward/).
90. National Autistic Society, "Diverse perspectives: The challenges for families affected by autism from Black, Asian and Minority Ethnic communities" (London, UK, 2014).
91. R. Trotter, "Over-looked Communities, Over-due Change: how services can better support BME disabled people" (London, UK, 2012).
92. S. Michie, M. M. van Stralen, R. West, *Implementation Science*. **6**, 42 (2011).
93. S. Michie *et al.*, *Annals of Behavioral Medicine*. **46**, 81–95 (2013).
94. J. Cane, D. O'Connor, S. Michie, *Implementation Science*. **7**, 1–17 (2012).
95. L. M. Collins, S. A. Murphy, V. N. Nair, V. J. Strecher, *Annals of Behavioral Medicine*. **30**, 65–73 (2005).
96. World Medical Association, *JAMA*. **310**, 2191–4 (2013).
97. NIHR / INVOLVE, "Involving children and young people as advisors in research: top tips and essential key issues for researchers" (2019), (available at https://www.invo.org.uk/posttypepublication/involving-children-and-young-people-as-advisors-in-research-top-tips-and-essential-key-issues-for-researchers/).
98. NIHR, Approvals, registration and governance | NIHR, (available at https://www.nihr.ac.uk/researchers/manage-your-funding/manage-your-project/approvals-registration-and-governance.htm).
99. Health and Care Professions Council, "Standards of conduct, performance and ethics" (London, 2016), (available at https://www.hcpc-uk.org/standards/standards-of-conduct-performance-and-ethics/).
100. U. G. Her Majesty's Stationary Office, *Data Protection Act 2018* (The Stationary Office, London, 2018).
101. NIHR, "Involving children and young people as advisors in research Top tips and essential key issues for researchers" (2021).
102. INVOLVE / NIHR, "Policy on payment of fees and expenses for members of the public actively involved with INVOLVE" (2016), (available at http://www.invo.org.uk/posttypepublication/national-institute-for-health-).
103. British Dietetic Association, "Code of Professional Conduct" (Birmingham, UK, 2017), (available at www.bda.uk.com).
